# Awareness and antibody detection of Newcastle disease virus in a neglected society in Nigeria

**DOI:** 10.14202/vetworld.2019.112-118

**Published:** 2019-01-21

**Authors:** Oluwafemi Babatunde Daodu, Julius Olaniyi Aiyedun, Rafiu Adebisi Kadir, Hauwa Motunrayo Ambali, Oladapo Oyedeji Oludairo, Isaac Dayo Olorunshola, Oluwakemi Christiana Daodu, Saka Saheed Baba

**Affiliations:** 1 Department of Veterinary Microbiology, Faculty of Veterinary Medicine, University of Ilorin, Nigeria, Sub Sahara Africa; 2 Department of Veterinary Public Health and Preventive Medicine, Faculty of Veterinary Medicine, University of Ilorin, Nigeria, Sub Sahara Africa; 3 Department of Veterinary Medicine, Faculty of Veterinary Medicine, University of Ilorin, Nigeria, Sub Sahara Africa; 4 Department of Wildlife and Ecotourism, Faculty of Agriculture, University of Ibadan, Nigeria, Sub Sahara Africa

**Keywords:** antibody detection, awareness, local birds, neglected communities, Newcastle disease, vaccination

## Abstract

**Aims:**

This study aimed to assess the level of awareness of rural poultry farmers on vaccination and to detect Newcastle disease virus (NDV) antibody in local birds (LB) and eggs in Kwara State, Nigeria.

**Materials and Methods:**

Data on farmers’ attitude, knowledge, practices, and experiences on ND mortality were obtained through an interview using a structured cross-sectional checklist. NDV antibodies were detected in sera and egg yolks of local chickens (LC) and guinea fowls (GF) using hemagglutination inhibition test.

**Results:**

A total of 83 interviewees, 287 sera and 121 egg yolk extracts, were examined. The study revealed that 98.8% (82/83) of the interviewee had never vaccinated their flock before. 90% of the interviewee had reported high mortality in birds within 1-6 months old, while the major clinical signs were cold (40.4%) and torticollis (30.8%). Evidences of LB exposure to wild-type NDV were confirmed by the detection of NDV antibodies in 20.8% and 0% of LC and GF, respectively. The mortality differences experiencedin <1 and 1-6 months old LB could be explained by the presence of maternally-derived NDV antibody (49.6%) in egg yolk.

**Conclusion:**

The study showed that LB suffers from NDV as a result of LB keepers’ ignorance and neglect by the government. This has limited local investment and subsequent contribution to gross domestic product. This study suggests that the key factors to the prevention of ND remain awareness creation about poultry vaccination, production of affordable vaccines, and availability/accessibility to veterinarian (or trained personnel).

## Introduction

Newcastle disease (ND) remains one of the most popular ravaging viral diseases limiting poultry production in Africa. It is caused by an RNA virus (ND virus [NDV]) of avian paramyxovirus designated as type 1 paramyxovirus which is a serotype under the genus *Avulavirus* of the family Paramyxoviridae [1]. The virus causes rapidly spreading, highly infectious nervous, respiratory, and gastrointestinal diseases in birds. Its severity depends on the viral factors (tropism and virulence), host factors (age, species, and immune status), and environmental factors (temperature, season, rainfall pattern, and relative humidity [2]. ND is endemic in many parts of the world and its economic pressure on poultry industry lingers [3,4].

Despite scientific achievements in its prevention by vaccination, neglected societies seem not to have benefitted from this success. Naturally, at the early stage of life, NDV maternally-derived antibody (MDAs) offer protection to chicks; however, the waning NDV MDAs result in susceptibility to wild-type NDV [5]. The advent of vaccination against NDV was a succor, yet despite the use of various NDV vaccine types (HB1, Lasota, and Komarov NDV vaccines) in commercial poultry farms, neglected communities (majorly rural and semi-urban communities) where local birds (LBs) are mostly raised continually experience high mortality caused by series of NDV outbreaks [4]. These neglected societies, which are populated by low-income earners, are still leaving in Dark Age before the scientific discovery of vaccine and drug. Initially, vaccines which require cold chains and administration expertise were developed, but in recent time, cheap thermostable vaccine with less expertise requirements is now available. One of these vaccines is NDV I-2 vaccine [6], and its use has been advocated for in rural poultry production [6,7]. Yet there seems not to be a continental spread of positive impact of this development on rural poultry production.

This study assessed the knowledge, attitude, and practices (KAP) of LB keepers on vaccination against NDV and its impact on poultry production in Kwara State, Nigeria. Exposure of LBs to wild-type NDV was also quantified by the presence of NDV antibodies in sera and egg yolks. This was done to correlate KAP in rural settings with our laboratory findings.

## Materials and Methods

### Ethical approval

All applicable International, National, and/or Institutional guidelines for the care and use of animals were duly followed.

### Informed consent

The consent of the live bird keepers were sort before the commencement of the interview. Only people who gave approval for an interview were included in the study.

### Study area

The study area was Kwara State. Interview was conducted in various local government areas (LGA) in Kwara State including Ilorin South LGA, Ilorin East LGA, Moro LGA, Irepodun LGA, and Ifelodun LGA. Local chickens (LCs) blood and eggs were obtained from Oja-titun poultry market located in Ilorin, Kwara State at latitude 8° 29’ 21.588” N and longitude 4° 31’ 53.8458” E. It is the major market which receives the largest number of LBs (of all ages) and eggs from all parts of Kwara State and other neighboring states for sale and/or slaughter and processing. Birds were kept based on species differences and fed in cages.

### Interview

A cross-sectional survey was conducted in rural and semi-urban areas in Kwara State where LBs are reared. The interview was conducted using English and/or indigenous language depending on the understanding of the interviewee. Interview of LB keepers was done using structured questions (checklist). The interview harvested data related to current KAP, challenges, and limitations of LB keepers that may limit or enhance successful and sustainable implementation of an ND vaccination program for LB. Commercial bird keepers and non-bird keepers were excluded from the interview.

### Bird selection and blood collection

A total of 287 blood samples were collected on a weekly basis from apparently healthy LCs (264) and guinea fowls (GFs) (23) at slaughter for dry (November 2015-January 2016) and wet seasons (March-August 2016). LBs were categorized into growers, hens, and cocks using weight (grower - ≥0.6 kg and hen/cock - >0.6 kg) and indigenous knowledge of the bird sellers. LCs sampled comprised growers (189), hens (54), and cocks (21). The samples were transported in a cold pack to the microbiology laboratory of the University of Ilorin Veterinary Teaching Hospital. The blood was allowed to clot and after that centrifuged at 2500 rpm for 10 min to separate the serum which was dispensed into well-labeled cryovial tube and stored at −20°C until the hemagglutination inhibition (HI) test was done.

### Egg yolk extraction

A total of 121 LC eggs were obtained for NDV antibody detection. 4 ml of egg yolk was mixed with 4 ml of phosphate-buffered saline in a sterile test tube. 2 ml of this mixture was then added to 4 ml of chloroform and incubated at room temperature for 1 h. The supernatant was separated into sterile cryovial tube after centrifugation of the mixture at 3000 rpm for 10 min. The sample was stored at −20°C until the use for HI test.

### Hemagglutination (HA) test and HI test

HA and HI tests followed procedures described in the OIE reference manual [3]. ND viral antibody was detected in sera and chloroform extracts of egg yolk.

### Statistical analysis

Qualitative data (interview) were summarized ethnographically and categorized according to the checklist. Key phrases were quoted to reflect participant’s views, beliefs, and perceptions about factors affecting the vaccination of local poultry.

The NDV antibody titer data were recorded as reciprocals of the highest dilution that caused HI, which was then logarithmically transformed, by log_2_ and all the analyses were done on the transformed data. HI titer (Log_2_) for each variable was calculated as geometric mean titers (GMTs). The percentage of positive sera was also calculated. Data were entered into GraphPad Prism version 5.03 (GraphPad Software Inc.) to test for significant differences in season and bird category using Fisher’s exact test and Chi-square test, respectively, at p=0.05.

## Results

### Interview

**Figure-1:**
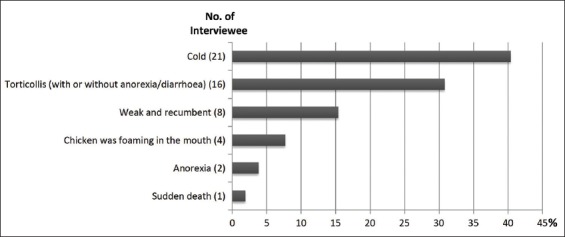
Percentage of clinical sign(s) shown before death. Percentage was calculated based on the number of interviewee who had experienced mortality.

**Figure-2:**
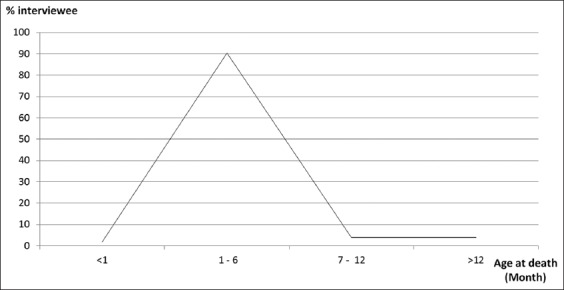
Percentage of age (month) of the chicken(s) at the point of death. Percentage was calculated based on the number of interviewee who had experienced mortality.

A total of 83 participants who keep LBs were interviewed. The interview revealed that 69.9% and 2.4% of the participants keep ≤10 and ≥41 chickens, respectively (Table-1). Cold (40.4%) and torticollis (30.8%) among other clinical signs were seen by 52 (62.7%) interviewee who experienced mortality in their flock (within a year) (Figure-1). Majority of the chickens were said to be within 1-6 months old before death (90%; 47/52 participants) (Figure-2).

**Table-1:** Awareness on village poultry vaccination and experience of keepers on ND.

Question/response	Frequency (%)
Present number of chicken owned	
1-10	58 (69.9)
11-20	18 (21.7)
21-30	3 (3.6)
31-40	2 (2.4)
≥41	2 (2.4)
Experience of chicken mortality within a year	
Yes	52 (62.7)
No	31 (37.3)
Treated chicken flock with commercial drug before	
Yes	26 (31.3)
No	57 (68.7)
Heard of vaccination in chicken	
Yes	21 (25.3)
No	62 (74.7)
^a^Reason(s) for not vaccinating chicken flock	
Lack of money	9 (42.9)
Availability of doctors and lack of fund	4 (19.0)
Non-availability of doctor	3 (14.3)
Have no deep understanding about chicken vaccination	2 (9.5)
Lack of money and time	1 (4.8)
They are local birds	1 (4.8)
None of them die	1 (4.8)
Done vaccination on my flock against diseases	
Yes	1 (1.2)
No	82 (98.8)
Most people I know would approve having my chickens vaccinated against ND at least every 6 months?	
Yes	45 (54.2)
No	38 (45.8)
How difficult it would be to get things needed for the services of a veterinarian (or other appropriate person) to vaccinate your chickens against ND at least every 6 months	
Very difficult	36 (43.4)
Somewhat difficult	19 (22.9)
Not difficult at all	28 (33.7)
How difficult it would be to remember to vaccinate my chickens against ND at least every 6 months	
Very difficult	24 (28.9)
Somewhat difficult	17 (20.5)
Not difficult at all	42 (50.6)
The likelihood of many of my chicken flock getting sick or die of ND	
Very likely	29 (34.9)
Somewhat likely	22 (26.5)
Not likely at all	32 (38.6)
How serious it would be if many of my chicken flock got sick or died of ND	
Very serious	56 (67.5)
Somewhat serious	15 (18.1)
Not serious at all	12 (14.5)
Presence of community laws or rules in place that makes it more likely that I vaccinate my flock against ND?	
Yes	8 (9.6)
No	75 (90.4)
God approves of vaccinating chickens	
Yes	43 (51.8)
No	37 (44.6)
I do not know	3 (3.6)
Presence of cultural rules or taboos against vaccinating chicken flock ND in my community	
Yes	6 (7.2)
No	74 (89.2)
I do not know	3 (3.6)

a Percentage was calculated based on the number of interviewee who had heard of vaccination in chicken, ND=Newcastle disease

**Figure-3:**
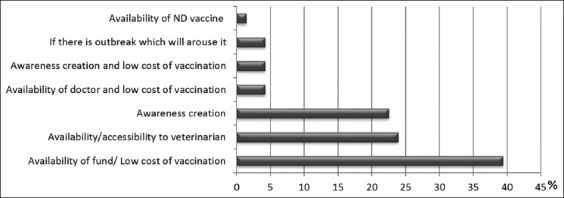
Factor(s) that support the feasibility of vaccination against Newcastle disease virus at least every 6 months.

Vaccination of chicken flock against infectious diseases (virus and bacteria) and the use of commercial poultry drugs were never practiced by 98.8% (82/83) and 68.7% (57/83) of the interviewee, respectively. Unawareness of poultry vaccinations was the main explanation given (74.7%, n=62) by ignorant poultry keepers (Table-1) while financial incapability was the key reason alluded by those who knew about poultry vaccination (n=9) (Figure-3). Among other factors, “availability of fund/low cost of vaccination” (39.4%), “availability/accessibility to veterinarian” (23.9%), and “awareness creation” (22.5%) were suggested to be essential for the feasibility of vaccination against NDV at least every 6 months (Table-1).

Thirty-eight (45.8%) interviewee asserted that most people they know would not approve having their chickens vaccinated (Table-1). Although 42 (50.6%) participants confirmed it would be easy for them to remember when to vaccinate their flock, 36 (43.4%) interviewee claimed that preparatory measures to be done before the services of a veterinarian (or other appropriate persons) to vaccinate chickens would be very difficult. Based on experience, 29 (34.9%) pronounced that it is very likely that many of their chicken flock get sick or die of ND while 67.5% (56/83) of participants said that the outcome would be very serious socioeconomically.

In general, participants claimed that there is no community law or rule in place that makes it more likely to vaccinate chickens (n=75), no cultural rule or taboo against vaccinating them (n=74), and God approves of vaccinating chickens (n=43) (Table-1).

### NDV antibody prevalence

NDV antibody was detected in 55 chicken sera (HI titer ≥4 Log_2_) given a prevalence of 20.8% in chickens (Table-1) while no antibody was found in GF sera. Although the modal titer and GMT values for chickens were 5 and 8.1, respectively (Table-2), 79.2% of them had <1:16 titer. GMT value (8.5) and modal titer (9.0) were high during dry season (Table-2) than in wet season. Based on age group, cock had the highest GMT value of 9.5 with a modal titer of 11. There were significant differences for the presence of NDV antibody in bird type, season, and age group (Table-3). Maternally-derived NDV antibodies were detected in LC egg yolks with GMT±SD and modal titer values of 5.4±3.9 and 3, respectively (Chart-4). HI titer showed that 50.4% of the egg yolk had NDV maternal-derived antibody <4 Log_2_.

**Table-2:** Modal titer and percentage distribution of NDV antibody in local chicken and guinea.

Features	Number of positive sample/total sample	Modal titer	Percentage ≥1:16 titer (%)	Percentage ≥1:128 titer (%)	GMT
Bird type					
Chicken	55/264	5	20.8	13.6	8.1
Guinea fowl	0/23	2.0	0.0	0.0	2.0
^a^Season					
Dry	44/161	9.0	27.3	21.1	8.5
Wet	11/103	5.0	10.7	1.9	6.2
^a^Bird categories					
Hen	4/54	4.0	7.4	0.0	4.8
Cock	4/21	11.0	19.0	19.0	9.5
Grower	47189	5.0	24.9	16.9	8.1

a Only local chickens were considered, GMT=Geometric mean titers

**Table-3:** Distribution of NDV antibody titers in local chicken and guinea fowl.

Features	Number of sera sample	Number of positive sample (%)	OR (95% CI)	p-value
Bird type				
Chicken	264	55 (20.8)	12.5 (*0.7-208.4)	0.0106
Guinea fowl	23	0 (0.0)	1	
^a^Season				
Dry	161	44 (27.3)	3.1 (1.5-6.4)	0.0011
Wet	103	11 (10.7)	1	
^a^Age group				
Hen	54	4 (7.4)	0.2 (0.1-0.7)	0.0043
Cock	21	4 (19.0)	0.7 (0.2-2.2)	0.7889
Grower	189	47 (24.9)	1	

* OR was calculated by adding 0.5 to each value,

a Only local chickens were considered, 95% CI=95% Confidence interval, OR=Odds ratio, NDV=Newcastle disease virus

## Discussion

ND is still an obscured epidemic among the rural communities in Nigeria where local poultry is one of the major sources of income and animal protein. These communities are neglected due to the advances disease prevention strategies applied in commercial poultry production. The ignorance about poultry vaccination against preventable infectious diseases (74%, n=62) and the use of drug(s) in control/treatment (68.7%, n=57) in this study depicted the reasons for the shortfall of LBs output in this disease endemic environment. This obliviousness had resulted in socioeconomic wastage. FAO [8] revealed that one of the major constraints to the achievement of an effective poultry disease control strategy is ignorance of poultry keepers. This study showed that the emotional trauma (67.5%; n=56) that might likely follow the loss of chickens to NDV would not encourage 69.9% of participants to keep >10 chickens as opposed to a large turnout in commercial poultry production. The economic impacts of livestock disease were described by Bennett [9], a reduction in the level of marketable outputs and its quality, waste of inputs, resource costs associated with disease prevention and control, human health costs associated with disease or disease control, negative animal welfare associated with disease, and international trade restrictions due to disease and its control. However, De Bruyn *et al*. [10] observed a bidirectional relationship, whereby ND vaccination led to greater chicken numbers, and larger flocks are more likely to be vaccinated. Thus, awareness and practice of vaccination against ND and other infectious diseases in village poultry can lead to a massive output as observed in this study.

The study revealed that torticollis (30.8%), a major sign of neurotropic strain of NDV, is the most frequently observed infectious clinical sign in LCs before death. However, MacLachlan and Dubovi [1] reported that the clinical signs associated with NDV in chickens are highly variable and this may include respiratory, circulatory, gastrointestinal, and nervous signs depending on the virus strain and host (age and immune status). The high frequency of cold and torticollis in this study might have resulted from ease to spot these clinical signs by LB keepers; other clinical signs of ND might have gone unnoticed.

**Figure-4:**
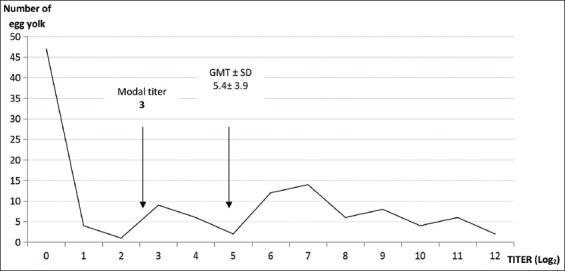
Newcastle disease virus hemagglutination inhibition titer of chloroform extracted egg yolk of local chickens.

The transfer of maternal NDV antibodies through the egg yolk to the intending chick was observed in this study (Figure-4). Unfortunately, the study indicated that 55.4% of chicks if hatched were likely to die from the lethal effect of wild-type NDV when exposed and unvaccinated. This was because no antibody was received from the hen through egg yolk (50.4% of egg yolk had HI titer <1:16). Furthermore, a sharp increase in mortality (highest peak) described among chickens between 1 and 6 months old (90.4%; n=47 participants) (Figure-2) suggested that maternally-derived NDV antibody offered protection to the chicks up to a month against wild-type NDV.

Although this study revealed that 45.8% of interviewee asserted that people around them would not approve them spending money on the vaccination of their chickens, the study suggests that the key factors to the prevention of ND through vaccination remain awareness creation about poultry vaccination, affordable vaccine, and availability/accessibility to veterinarian (Table-1). This is in agreement with the report of FAO [8] which listed these as constraints that have to be defeated for a comprehensive poultry disease control and prevention in any neglected society.

The presence of NDV antibodies in LBs (prevalence=20.8%) and their eggs (prevalence=44.6%) in this study confirmed that the birds were exposed to wild-type NDV since they were never vaccinated. This strengthens previous reports of the endemic nature of NDV in Nigeria [11,12]. Furthermore, in this study, 79.2% of LCs possessed <1:16 HI titer and might develop ND, whenever there is an outbreak.

It is imperative to suggest that most LCs obtained from rural communities and some urban areas in this study area were survivors of infectious diseases, of which NDV played a prominent role since they were unvaccinated but yet exposed in NDV endemic environment (Tables-1-3 and Figure-2) [12,13].

The occurrence of high HI-positive sera in LCs (20.8%) and none in GF in this study can be compared with Boakye *et al*. [13] who reported high positive sera in LCs (81.8%) than in GFs (24.2%) in area of Kumasi, Ghana. It has been reported that chickens are highly susceptible to velogenic strain of NDV while gallinaceous birds such as pheasants, GF, partridges, peacocks, and quails have variable susceptibility [14]. In fact, GF can carry velogenic strain subclinically [14]. Furthermore, the prevalence of 20.8% LCs possessing NDV antibody was higher than 17% reported in Federal Capital Territory of Nigeria which is in the same region (Northcentral) of the study area [12]. In this study, chickens were 12.5 more likely to have been exposed to NDV compared to GF at a significant difference of p=0.0016 (95% confidence interval [CI]=0.7-208.4).

Furthermore, this study showed that chickens were 3.1 times more exposed to wild-type NDV during dry season (27.3%; n=44/161) compared to wet season (10.7%, 11/103) and this was significant (p=0.0011; 95% CI=1.5-6.4). LCs which are generally managed under free-range and backyard systems [15] are likely to scavenge more intensely during the dry season due to feed scarcity and thus are exposed to NDV which is endemic in the environment. Furthermore, increasing amount of dust, wind velocity, varying temperature, and stress could reduce the efficiency of immune system to respond to infection, thus might attribute to high NDV infection during the dry season. This difference could also explain the seasonal shortage of the supply of LCs during dry period [16]. This might culminate from increasing mortality in the chicken flock leaving out the survivors for the market. Furthermore, this study indicated that hen and cock were 0.8 and 0.3 times less likely to be exposed to wild-type NDV respectively when compared to grower chickens. A significance difference (p=0.0043) was found when the presence of NDV antibodies in hen and grower was compared.

The significant differences to the exposure of LBs to wild-type NDV in season and in bird group further suggest needs for deeper investigation for a holistic solution to challenges in LC production in semi-urban and rural communities.

The absence of cultural rule or taboo against vaccination in the study area is likely to ease acceptability and practice. Furthermore, education of local poultry keepers should be done with adequate knowledge of traditional beliefs, and this is likely to yield a huge positive response toward LB vaccination against NDV and other infectious diseases.

## Conclusion

This study showed that vaccination against preventable poultry diseases was not practiced by LB keepers due to so many factors, of which unawareness, financial incapability, and inaccessibility to veterinarian (or other qualified personnel) remain prominent. Furthermore, the study showed that LBs in the study area were exposed to wild-type NDV and resulted in mortality among the flocks. This suggested the reasons for low production output of LBs which are themselves survivors. The maternally-derived NDV antibodies were also detected in the eggs of LBs and can serve as a guide for vaccination protocol design during mass vaccination in local poultry production in Nigeria.

## Authors’ Contributions

OBD and OCD conceived and designed the research study. OBD, OCD, JOA, RAK, and HMA collected the sample and conducted the interview. OBD, JOA, and RAK carried out the laboratory assays while OBD, JOA, and OOO analyzed data. OBD, JOA, IDO, and SSB drafted the manuscript. All authors read, revised, and approved the final manuscript.
